# Mind-body internet and mobile-based interventions for depression and anxiety in adults with chronic physical conditions: A systematic review of RCTs

**DOI:** 10.1371/journal.pdig.0000435

**Published:** 2024-01-23

**Authors:** Emily Johnson, Shaina Corrick, Serena Isley, Ben Vandermeer, Naomi Dolgoy, Jack Bates, Elana Godfrey, Cassidy Soltys, Conall Muir, Sunita Vohra, Puneeta Tandon

**Affiliations:** 1 Division of Gastroenterology (Liver Unit), University of Alberta, Edmonton, Alberta; 2 Department of Medicine, University of Alberta, Edmonton, Alberta; 3 Faculty of Rehabilitation Science, Edmonton, Alberta; 4 Faculty of Science, University of Alberta, Edmonton, Alberta; 5 Faculty of Science, University of Toronto, Toronto, Ontario; 6 Department of Pediatrics, University of Alberta, Edmonton, Alberta; Iran University of Medical Sciences, IRAN (ISLAMIC REPUBLIC OF)

## Abstract

This review summarizes the effectiveness of scalable mind-body internet and mobile-based interventions (IMIs) on depression and anxiety symptoms in adults living with chronic physical conditions. Six databases (MEDLINE, PsycINFO, SCOPUS, EMBASE, CINAHL, and CENTRAL) were searched for randomized controlled trials published from database inception to March 2023. Mind-body IMIs included cognitive behavioral therapy, breathwork, meditation, mindfulness, yoga or Tai-chi. To focus on interventions with a greater potential for scale, the intervention delivery needed to be online with no or limited facilitation by study personnel. The primary outcome was mean change scores for anxiety and depression (Hedges’ *g*). In subgroup analyses, random-effects models were used to calculate pooled effect size estimates based on personnel support level, intervention techniques, chronic physical condition, and survey type. Meta-regression was conducted on age and intervention length. Fifty-six studies met inclusion criteria (sample size 7691, mean age of participants 43 years, 58% female): 30% (n = 17) neurological conditions, 12% (n = 7) cardiovascular conditions, 11% cancer (n = 6), 43% other chronic physical conditions (n = 24), and 4% (n = 2) multiple chronic conditions. Mind-body IMIs demonstrated statistically significant pooled reductions in depression (*SMD* = -0.33 [-0.40, -0.26], p<0.001) and anxiety (*SMD* = -0.26 [-0.36, -0.17], p<0.001). Heterogeneity was moderate. Scalable mind-body IMIs hold promise as interventions for managing anxiety and depression symptoms in adults with chronic physical conditions without differences seen with age or intervention length. While modest, the effect sizes are comparable to those seen with pharmacological therapy. The field would benefit from detailed reporting of participant demographics including those related to technological proficiency, as well as further evaluation of non-CBT interventions.

**Registration:** The study is registered with PROSPERO ID #CRD42022375606.

## Author summary

Depression and anxiety are common in people living with chronic physical conditions. These symptoms are associated with increased morbidity and reduced quality of life. Mind-body wellness techniques are gaining evidence as practical strategies to reduce these symptoms and improve quality of life across a range of chronic physical conditions. Internet and mobile platforms have become increasingly popular since the COVID-19 pandemic, allowing for delivery of the interventions in the convenience of a patient’s home and across geographical boundaries. The aim of this systematic review and meta-analysis was to examine the effectiveness of mind-body internet and mobile interventions (IMIs) including yoga, Tai-chi, breathwork, and cognitive behavioral therapy delivered with limited or no personnel support on symptoms of anxiety and depression relative to control conditions. The inclusion of IMIs as well as interventions with no or limited personnel facilitation uniquely focuses this review on scalable interventions. Recognizing the limitation of moderate heterogeneity, our findings indicate that these interventions can effectively reduce anxiety and depression symptoms in individuals living with chronic physical conditions when compared to controls, with an effect size similar to those seen with pharmacological therapy.

## Introduction

An estimated five billion people live with at least one chronic physical condition, with close to 30% of these individuals living with two or more conditions [1]. Defined by the World Health Organization as *“conditions requiring ongoing management and treatment over extended periods of time*” [2], chronic physical conditions account for an estimated 64% of Disability Adjusted Life Years lost each year globally [3]. These conditions are not only associated with socioeconomic consequences [4] and reduced quality of life [5], but also substantial comorbid mental health symptoms [6]. Systematic reviews have identified an average prevalence of depressive symptoms of 27% [7] and anxiety symptoms ranging from 11–80% depending on the chronic physical condition under evaluation [8]. With increasing recognition of the role of non-pharmacological options in the management of mental health symptoms, there have been a number of studies [9–12] supporting the impact of mind-body wellness techniques as effective management strategies [13].

Mind-body wellness is *“an approach that focuses on the interactions among the brain*, *mind*, *body and behavior”* [14]. Mind-body wellness techniques are based in the perspective that mental and physical health affect each other. These most commonly include techniques such as yoga, meditation and Tai-chi [14,15], but can also include psychotherapy based interventions such as cognitive behavioral therapy (CBT) [16–18]. Mind-body practices are becoming increasingly offered via websites or mobile applications herein referred to as internet and mobile-based interventions (IMIs). This allows reach to a broader group of people across geographic barriers [19,20]. However, despite several reviews reporting positive impacts of mind-body IMIs, these have been limited by the inclusion of: i) specific-mind body techniques (i.e., CBT only, yoga only) [21–24]; ii) specific chronic conditions [25–28]; iii) both chronic mental and physical health conditions despite unique etiologies and symptoms [29]; iv) healthy populations [23,30]; v) non-randomized trial designs [31] or vi) the inclusion of different levels of personnel-facilitation or mixture of in-person and online intervention delivery components within a single study [32]. The latter point is of importance as a high degree of personnel support or the requirement for an in-person delivery component to IMI interventions, while suitable for constrained research projects or high-risk patients, can limit real-world applicability and scale. Across a range of chronic conditions, therefore, there remains uncertainty whether mind-body IMIs have a greater effect across intervention and participant characteristics (intervention length, personnel support level, age), as well as uncertainty about what harms have been identified, and how adherence data are collected and reported.

Accordingly, in individuals living with a range of chronic physical conditions, the primary aim of this review was to systematically review the literature on selected scalable mind-body IMIs (yoga, Tai-chi, breathwork, meditation, mindfulness, CBT, or CBT derivatives) in order to improve the understanding of their effect on symptoms of anxiety and depression. To achieve this, the objectives of this review were to (i) assess the effect of mind-body IMIs on symptoms of anxiety and depression evaluated using psychometrically validated questionnaires in the context of randomized controlled trials RCTs), (ii) to understand the impact of different mind-body techniques, chronic physical condition type, level of personnel support, type of survey used, participant age, and intervention length on symptoms of anxiety and depression, and (iv) to summarize how studies gathered and reported harms and study and intervention adherence data. Based on data from published studies, it was hypothesized that mind-body IMIs would demonstrate significant benefit irrespective of the chronic physical condition type [6,33]. It was hypothesized that more benefit would be seen with a longer intervention duration that would allow for more time to fully engage with intervention content [34,35], and with increased personnel support that would support accountability [36,37]. Increased benefit was also anticipated with younger participants, as they may present with higher levels of psychological distress [38], and may also find it easier to engage with online interventions due to higher digital technology proficiency [39].

## Methods

### Search strategy

Electronic searches were independently performed in PsycINFO, MEDLINE, EMBASE, CINAHL, Cochrane CENTRAL, and SCOPUS (last update March 17, 2023). The search strategy included any RCT of mind-body IMIs in people living with chronic physical conditions with anxiety and depression outcomes. The search for all databases is presented in the S1 Appendix. An English language restriction was imposed. In addition, hand searches were conducted among reference lists of relevant review articles.

### Study selection

Articles retrieved during the searches were screened for relevance; those considered as potentially eligible were evaluated based on the inclusion/exclusion criteria outlined using PICOD [40]. *Population*–Inclusion required adult (≥18 years of age) participants living with a chronic physical condition. Studies recruiting participants living with chronic primary mental health conditions (e.g., major depressive disorder), involving participants described as ‘cancer survivors’ or involving pediatric patients or their caregivers were excluded. *Intervention*–Interventions delivering one of the three categories of mind-body wellness techniques targeted in this review: “CBT” (includes CBT or CBT derivatives), “non-CBT” (includes selected non-CBT mind-body wellness techniques ‐ breathwork, meditation, mindfulness, yoga, Tai-chi), and “CBT+" (includes techniques from both CBT and non-CBT categories). Interventions had to be delivered through internet or mobile platforms, excluding digital video discs (DVD) and teleconference methods. They could be either "self-guided" without study personnel support or "personnel facilitated" which involved personnel to support participation. This excluded those interventions in which personnel were required to deliver the intervention (e.g., personnel delivering a weekly CBT therapy session). *Comparator*–No intervention or intervention not containing a mind-body technique. *Outcomes*–Included studies required pre-and-post anxiety and/or depression questionnaires that were psychometrically validated. *Design*–Only RCT studies were included.

### Data abstraction

Articles returned were imported into the Covidence review management system [41]. References were examined at the title/abstract level independently by two authors and, if potentially suitable for inclusion, were retrieved as complete articles. One author extracted data and a second author verified the extracted data using a form constructed on REDCap [42]. The primary outcome was the difference in mean scores of validated depression and anxiety measures with the associated 95% confidence intervals (CIs). For studies that used multiple surveys to measure anxiety or depression, the Hospital Anxiety and Depression Scale (HADS) was prioritized for the primary analysis, followed by the Personal Health Questionnaire-8/9 (PHQ-8/9) for depression, and Generalized Anxiety Disorder-7 (GAD-7) for anxiety. These surveys were chosen post-hoc based-on frequency of scale use. Secondary outcomes were comparisons of mind-body technique, chronic physical condition type, personnel support level, and description of study and intervention adherence data. Demographic characteristics, including sex, gender, race, technological literacy, and age were also collected. If reported, intention-to-treat data were used. Harms data was collected, including if adverse events were reported, the frequency within each study arm, and how this information was collected. In circumstances of missing or unclear data, an email attempt to contact the corresponding author was made.

### Risk of bias assessment

The risk of bias in each study was assessed by two authors using the revised Cochrane risk-of-bias tool for RCTs (version 2.0) [43]. Each of the five risk domains was scored against a three-point rating scale, corresponding to a low, moderate, and high risk of bias.

### Data analysis and synthesis

This systematic review and meta-analysis was registered on PROSPERO (CRD42022375606) and follows the Preferred Reporting Items for Systematic Reviews and Meta-Analyses (PRISMA) guidelines [44]. All analyses were performed with ReviewManager (version 5.3) and Stata 17 [45,46]. Data were synthesized using random effects based on Hedges’ *g* statistic, which is used to estimate the effect size for the difference between means of continuous measures between the intervention and control conditions. Pooled standardized mean differences (SMDs) and their 95% CIs were estimated for depression and anxiety outcomes to allow a comparison of all studies together regardless of the use of different scales. Calculations for missing variables (i.e., missing mean change scores) were completed in accordance with “Handling Continuous Outcomes in Quantitative Synthesis: Methods Guide for Comparative Effectiveness Reviews” [47]. For baseline calculations in which no correlation was provided, a correlation of 0.5 was imputed. For manuscripts with multiple follow-up points, the collection points closest to the completion of the intervention were used. In cases where intervention length was not defined by the authors, the mean completion length by participants were used. Statistical significance was set at the two-tailed 0.05 level for hypothesis testing. Unadjusted p-values are reported throughout. As per previous recommendations, an effect size of 0.2 was determined as small, 0.5 medium as medium, and 0.8 as large [23]. Heterogeneity was quantified using the I^2^ statistic [48]. Results from studies grouped according to pre-hoc study level characteristics were compared using random effects meta-regression (age, intervention length) or stratified meta-analysis (subgroups by survey type, mind-body technique, chronic physical condition group, personnel facilitation versus self-guided).

## Results

### Study selection

**Fig 1:**
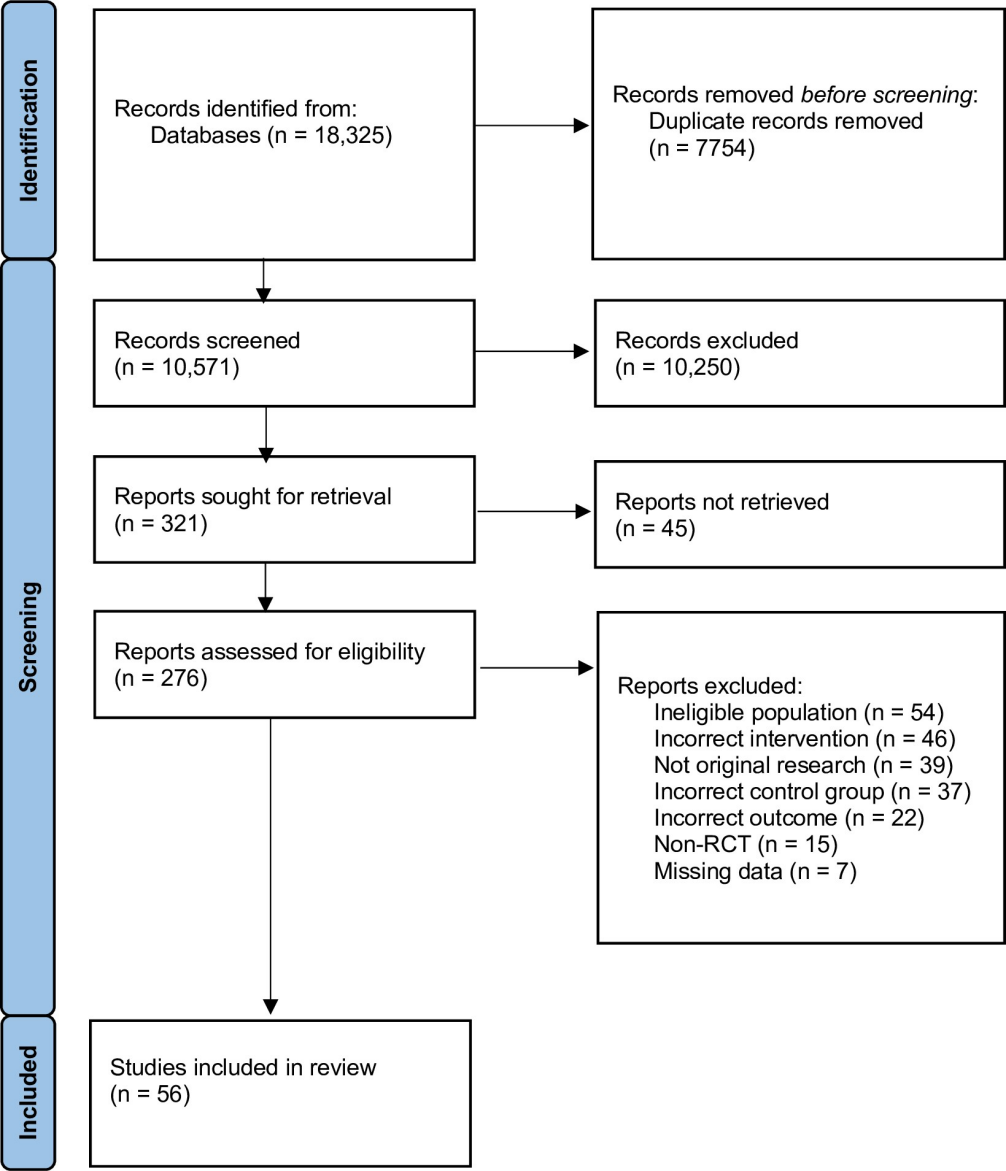
PRISMA Flow Diagram.

Database screening and manual searches yielded a total of 18,325 articles; 56 RCTs met inclusion criteria, involving a total of 7691 study participants (**Fig 1**).

### Study characteristics

The mean age of participants was 43 years (ranged from 18–86 years) and the mean number of participants per study was 137 (ranged from 20–676). Most participants were female (n = 4620 (58%)), with one study including only males [49], one including only females [50], and one not reporting participant sex [51]. Most were conducted in the United States (10, 18%) [52–61], Sweden (9, 16%) [62–70], Netherlands (8, 14%) [71–78] and the United Kingdom (9, 16%) [49,51,79–85] with the remaining 20 trials conducted in other countries. Twenty-four different chronic physical conditions were identified; for ease of analysis the conditions were divided into four post-hoc groupings based on frequency: neurological conditions (17, 30%) (e.g., Parkinson’s disease) [52,53,59,62,63,66,71,80,82,83,85–91] followed by cardiovascular conditions (e.g., heart failure) (7, 12%) [65,69,70,74,78,92,93], cancer (6, 11%) [54,57,58,64,94,95], and other (24, 43%) (e.g., HIV). Two trials enrolled participants living with multiple chronic conditions [96,97].

Included trials (**Table 1**) were two-armed RCTs (55, 98%) and three-armed RCTs (1, 2%). Control groups either received no intervention (22, 39%) [54,59,61,64,70,72,73,75,77,78,82,85,90,93,95,97–103], basic education (pamphlet, online information) (2, 4%)[79, 84], access to a discussion forum (5, 9%) [63,65,67–69], or were waitlisted and received treatment as usual (27, 48%) [49–58,60,62,66,71,76,80,81,83,86–89,91,94,96,104]. Interventions lasted between 4 weeks and 12 months (median = 9 weeks). The interventions were predominantly delivered using a website (49, 88%) [49,50,52–54,56,59–78,80–94,96–101,104,105], with the minority delivered on mobile applications (5, 9%) [51,55,58,95,103], or a combination (2, 3%)[57,79]. Nine interventions (16%) [49,51,57,58,87,88,91,95,103] were commercially available (e.g., Headspace) and the remainder were created specifically for the research study. The most commonly used mind-body wellness intervention was CBT (28, 50%) [56,59,61,63–66,69–75,77,80–83,85,92,93,96–100], followed by interventions with a combination of CBT and non-CBT techniques (CBT+) (18, 32%) [49,50,52,53,55,62,67,68,76,84,86,87,89–91,94,101,105], and then non-CBT techniques alone (10, 18%) [51,54,57,58,60,78,79,95,103,104]. Most studies assessed both anxiety and depression (47, 84%) with three studies (5%) reporting only on anxiety [56,61,67] and six studies (11%) reporting only on depression [65,82,87,91,100,104]. Seventy-three percent (n = 41) of studies provided mental health specific inclusion/exclusion criteria. Nineteen of these studies used specific thresholds on mental health questionnaires to determine eligibility; 8 for inclusion [49,65,69,73,75,77,89,93], 5 for exclusion [54,55,64,96,99], and 6 for both inclusion and exclusion [70,71,76,82,92,105]. Sixteen studies specifically named suicidality as an exclusion criteria [55,63,64,66,67,69–71,76,87,88,91,92,96,99,100]. A similar number of studies included interventions that were either “self-guided” (25, 45%) [49,51,54,55,57,58,60–62,78,79,81,82,85,87–91,94,95,98,100,101,104] or included “personnel-facilitation” (31, 55%) [50,52,53,56,59,63–73,75–77,80,83,84,86,92,93,96,97,99,102,103,105]. Studies described as “self-guided” were interventions that ranged from no support to, at most, technology support from study personnel to ensure participants were able to access the intervention. Studies described as “personnel facilitated” ranged from infrequent emails or communication through the digital platform with study personnel, to at most, weekly brief communication (∼15 minutes) via video, phone, or email to give feedback on homework assignments.

**Table 1:** Key Study Characteristics.

Author (Year)	Country	Total N	Intervention Type	Intervention Duration	Condition	Outcomes Measured*	Measure(s)	Personnel support level	Mental health inclusion/exclusion criteria
Ainsworth 2019[79]	United Kingdom	88	Non-CBT	< 12 weeks	Other (Asthma)	D, A	HADS	Self-guided	None
Ainsworth 2022[51]	United Kingdom	158	Non-CBT	12 weeks	Other (Asthma)	D, A	HADS	Self-guided	Inclusion: None Exclusion: previous diagnosis of major or unstable comorbid psychological disorders, other than anxiety or depression
Andersson 2002[62]	Sweden	117	CBT+	6 weeks	Neurological	D, A	HADS	Self-guided	None
Bendig 2021[92]	Germany	34	CBT	8 +/- 4 weeks	Cardiovascular	D, A	PHQ-9; GAD-7	Personnel-facilitated Personnel: Master level psychology students Facilitation: Standardized message through the IMI platform based on module completion. Option to receive reminders/motivational messaging	Inclusion: Depressive symptoms (PHQ ≥ 5) Exclusion: increased suicidality (PHQ item 9 [thoughts that you would rather be dead or want to harm yourself] on more than half of the days or more); lifetime diagnosis of psychotic, schizophrenia, or bipolar disorder
Beukes 2018[80]	United Kingdom	146	CBT	8 weeks	Neurological	D, A	PHQ-9; GAD-7	Personnel-facilitated Personnel: Audiologist supervised by CBT-psychologist Facilitation: Assignment feedback, support, and motivational messaging through the IMI platform	Inclusion: None Exclusion: Reporting any major medical, psychiatric, or mental disorder, which may hamper commitment to the program.
Boeschoten 2016[71]	Netherlands	171	CBT (Problem solving therapy)	5 weeks	Neurological	D, A	HADS	Personnel-facilitated Personnel: Masters level psychology students Facilitation: Optional communication through IMI platform to support use of intervention	Inclusion: BDI score of at least 14 on two consecutive occasions and no treatment from psychologist, psychotherapist or psychiatrist within the last three months. Exclusion: BDI score of at least 29 on two consecutive occasions; active suicidal ideas; current or life-time diagnosis of psychosis, organic mental disorder or substance dependency
Bromberg 2011[52]	United States of America	185	CBT+	4 weeks	Neurological	D, A	DASS-21	Personnel-facilitated Personnel: Research team Facilitation: Email communication with module instructions/checklists to keep on track	None
Bundy 2013[81]	United Kingdom	126	CBT	6 weeks	Other (Psoriasis)	D, A	HADS	Self-guided	Inclusion: None Exclusion: If they suffered a current psychiatric illness, were receiving psychological treatment
Burke 2019[86]	Ireland	69	CBT+	6 weeks	Neurological	D, A	HADS	Personnel-facilitated Personnel: Physiotherapist + research team Facilitation: One-time webinar and optional phone line	Inclusion: None Exclusion: Mental health issues requiring active psychiatric management; substance misuse
Cardol 2023[72]	Netherlands	121	CBT	15 weeks	Other (Chronic kidney disease)	D, A	PHQ-9; GAD-7	Personnel-facilitated Personnel: Health psychologist Facilitation: Initial intake interview followed by weekly/biweekly messaging on the IMI platform	Inclusion: None Exclusion: Medical conditions that are likely to interfere with study completion (e.g., progressive malignancy, recent cardiovascular event, severe psychiatric disorders) at the discretion of the nephrologist
Cooper 2011[82]	United Kingdom	24	CBT	8 weeks	Neurological	D	BDI	Self-guided	Inclusion: BDI score of at least 14 on two consecutive occasions and no treatment from psychologist, psychotherapist or psychiatrist within the last three months. Exclusion: BDI score of at least 29 on two consecutive occasions; active suicidal ideas; current or life-time diagnosis of psychosis, organic mental disorder or substance dependency
Dear 2022[96]	Australia	676	CBT	8 weeks	Multiple conditions	D, A	PHQ-9; GAD-7	Personnel-facilitated Personnel: Registered/clinical psychologists Facilitation: Weekly text messages or phone call	Inclusion: Reported that their health condition had a significant impact on their emotional well-being and quality of life Exclusion: If they reported very severe symptoms of depression (i.e., scoring >24 on the PHQ-9) or if they reported suicide plans or a recent suicide attempt
Devineni 2004[53]	United States of America	86	CBT+	4 weeks	Neurological	D, A	CES-D; STAI	Personnel-facilitated Personnel: Research team Facilitation: Optional email support. Weekly progressive muscle relaxation group session.	None
Drozd 2014[104]	Norway	67	Non-CBT	5 weeks	Other (HIV)	D	CES-D	Self-guided	None
Ferwerda 2017[73]	Netherlands	133	CBT	26 weeks**	Other (Rheumatoid arthritis)	D, A	BDI; IRGL	Personnel-facilitated Personnel: Clinical psychologist Facilitation: Feedback on homework through the IMI platform	Inclusion: Elevated levels of distress as measured by heightened scores of the negative mood and anxiety scales of the Impact of Rheumatic Diseases on General Health and Lifestyle (IRGL) (scoring equal or higher than 5 on the negative mood scale (6 items, range 0–24) or equal or higher than 21 on the anxiety subscale (10 items, range 10–40)) Exclusion: Severe physical or psychiatric comorbidity (i.e., physical or psychiatric comorbidity which required acute and/or intensive medical attention or which was a more impacting condition than rheumatoid arthritis according to the patient view); current treatment by a cognitive-behavioral therapist or comparable practitioner
Fischer 2015[87]	Germany	90	CBT+	9 weeks	Neurological	D	BDI	Self-guided	Inclusion: Self-reported depressive symptoms Exclusion: Diagnosed with bipolar or schizophrenia spectrum disorders, had substantial neurocognitive impairment such as dementia, or had suicidal ideations
Habibovic 2014[74]	Netherlands	289	CBT	12 weeks	Cardiovascular	D, A	HADS	Personnel-facilitated Personnel: Masters level psychologists Facilitation: Feedback on homework through the IMI platform	Inclusion: None Exclusion: History of psychiatric illness other than affective/anxiety disorders
Hauffman 2020[64]	Sweden	245	CBT	10 weeks	Cancer	D, A	HADS	Personnel-facilitated Personnel: Psychologists Facilitation: Feedback on homework through the IMI platform	Inclusion: None Exclusion: Severe depression or suicide risk on the MADRS-S
Hesser 2012[63]	Sweden	99	CBT	10 weeks	Neurological	D, A	HADS	Personnel-facilitated Personnel: Therapists Facilitation: Optional messaging through the IMI platform	Inclusion: None Exclusion: Severe medical or psychiatric condition, presented with an imminent suicide risk
Hilmarsdottir 2021[103]	Iceland	30	Non-CBT	24 weeks	Other (Diabetes)	D, A	HADS	Personnel-facilitated Personnel: Research team Facilitation: Motivational messaging through the IMI platform	None
Huberty 2019[54]	United States of America	48	Non-CBT	12 weeks	Cancer	D, A	ED-ASF 8a/ED-DSF 8a	Self-guided	Inclusion: None Exclusion: had a score of ≥15 on the PHQ-9
Hunt 2009[56]	United States of America	121	CBT+	8 weeks	Other (Irritable bowel syndrome)	D, A	PHQ-9; DASS-21	Self-guided	None
Hunt 2021[55]	United States of America	54	CBT	5 weeks	Other (Irritable bowel syndrome)	A	ASI	Personnel-facilitated Personnel: Therapist Facilitation: Feedback on homework via email	Inclusion: None Exclusion: Severe depression and/or suicidal ideation (defined as a score of 20 or higher on PHQ-9 and/or positive endorsement of active suicidal ideation or intent on a separate suicide question)
Johansson 2019[65]	Sweden	144	CBT	9 weeks	Cardiovascular	D	PHQ-9	Personnel-facilitated Personnel: Nursing staff with CBT training Facilitation: Feedback on homework through the IMI platform. Asynchronous messaging through platform	Inclusion: Suffered at least mild depressive symptoms (PHQ-9 score ≥5) Exclusion: Severe depression assessed as requiring acute treatment
Kraepelien 2020[66]	Sweden	77	CBT	10 weeks	Neurological	D, A	HADS	Personnel-facilitated Personnel: Therapists Facilitation: Weekly feedback on homework and motivational messaging via the IMI platform	Inclusion: None Exclusion: Psychotic disorder, Bipolar, or other serious psychiatric disorder; high suicide risk
Kubo 2019[58]	United States of America	97	Non-CBT	8 weeks	Cancer	D, A	HADS	Self-guided	Inclusion: None Exclusion: Severe mental illness
Kubo 2020[57]	United States of America	103	Non-CBT	6 weeks	Cancer	D, A	HADS	Self-guided	None
Lee 2019[50]	Taiwan	128	CBT+	6 weeks	Other (Irritable bowel syndrome)	D, A	CES-D; STAI	Personnel-facilitated Personnel: Therapists Facilitation: Daily feedback on homework through the IMI platform	None
Ljotsson 2010[67]	Sweden	61	CBT+	10 weeks	Other (Irritable bowel syndrome)	A	VSI	Personnel-facilitated Personnel: Graduate psychology student Facilitation: Asynchronous message via the IMI platform	Inclusion: None Exclusion: Suicidal ideation, severe depressive symptoms, substance dependence, psychosis, manic episode
Ljotsson 2011[68]	Sweden	86	CBT+	10 weeks	Other (Irritable bowel syndrome)	D, A	MADRS-S; VSI	Personnel-facilitated Personnel: Clinical therapists Facilitation: Weekly message via the IMI platform and feedback on homework	Inclusion: None Exclusion: Were judged to be highly unsuitable for iCBT for somatic or psychological reasons
Lundgren 2016[69]	Sweden	50	CBT	9 weeks	Cardiovascular	D, A	PHQ-9; CAQ	Personnel-facilitated Personnel: Mental health nurses with experiences in heart failure Facilitation: Feedback on homework via the IMI platform.	Inclusion: Mild depressive symptoms (PHQ-9 ≥5) Exclusion: Suffering from severe disease or illness that hindered participation in study; severe depressive symptoms; high level of suicide risk; other psychiatric disorder that makes intervention unsuitable
McCombie 2016[101]	New Zealand	231	CBT+	8 weeks	Other (Inflammatory bowel disease)	D, A	HADS	Self-guided	Inclusion: None Exclusion: Existing psychotic disorder or in psychotherapy; substance dependent
Mensorio 2019[98]	Spain	106	CBT	9 weeks	Other (Obesity)	D, A	DASS-21	Self-guided	None
Meyer 2018[88]	Germany	200	CBT	24 weeks	Neurological	D, A	PHQ-9; GAD-7	Self-guided	Inclusion: Diagnosis of depressive disorders; moderate depression Exclusion: Antidepressant medication newly prescribed or changed within one month; currently in psychotherapy; diagnosed with bipolar; schizophrenia, other psychotic disorder, borderline personality disorder; acute suicidality
Migliorini 2016[89]	Australia	59	CBT+	11 weeks**	Neurological	D, A	DASS-21	Self-guided	Inclusion: Scored above normative threshold of DASS-21 Exclusion: None
Moss-Morris 2012[83]	United Kingdom	45	CBT	10 weeks	Neurological	D, A	HADS	Personnel-facilitated Personnel: Assistant psychologist Facilitation: Three phone calls (30–60 mins) through the course of the program to share feedback and discuss content.	None
Motl 2017[59]	United States of America	47	CBT	24 weeks	Neurological	D, A	HADS	Personnel-facilitated Personnel: Behavioral therapists Facilitation: One to one short video chats throughout the 6-month program to discuss content and encourage adherence	None
Murray 2017[84]	United Kingdom	374	CBT+	12 months	Other (Type II diabetes)	D, A	HADS	Personnel-facilitated Personnel: Diabetes practice nurse Facilitation: Initial orientation session followed by newsletter emails and text message reminders about events and new content	None
Nadort 2022[75]	Netherlands	191	CBT	10 weeks	Other (Chronic Kidney Disease)	D, A	BDI-II; BAI	Personnel-facilitated Personnel: Therapist Facilitation: Weekly feedback on homework via the IMI platform	Inclusion: Increased levels of depressive symptoms (score of ≥10 on the BDI-II) Exclusion: Actively suicidal
Neubert 2023[94]	Germany	172	CBT+	4 weeks	Cancer	D, A	PHQ-8; GAD-7	Self-guided	Inclusion: None Exclusion: Severe mental impairments
Newby 2017[99]	Australia	106	CBT	10 weeks	Other (Type II diabetes)	D, A	PHQ-9; GAD-7	Personnel-facilitated Personnel: Masters or PhD-level clinical psychologist Facilitation: Two initial group lessons followed by motivational messages via email	Inclusion: Meet criteria for major depressive disorder according to telephone-administered diagnostic interview Exclusion: Had a self-reported diagnosis of bipolar affective disorder, psychotic disorder or substance use disorder, or were taking antipsychotics or benzodiazepines; had commenced CBT in the past month; changed antidepressant medication in the past 2 months; scoring either <5 (normal range) or >23 (very severe) on the PHQ-9; those identified as being at significant risk of suicide or deliberate self-harm in the telephone risk assessment
Norlund 2018[70]	Sweden	239	CBT	14 weeks	Cardiovascular	D, A	HADS	Personnel-facilitated Personnel: Licensed psychologist Facilitation: Feedback on homework via the IMI platform	Inclusion: Score >7 on one or both of the 2 HADS subscales Exclusion: Self-reported severe depression or suicidal ideation (MADRS-S total score >34 or MADRS-S item 9>3)
O’Moore 2018[100]	Australia	77	CBT	10 weeks	Other (Knee osteoarthritis)	D	PHQ-9	Self-guided	Inclusion: Met criteria for major depressive disorder (MDD) based on the clinician-administered Mini-International Neuropsychiatric Interview (MINI) Exclusion: Met criteria for bipolar, psychotic or substance dependent disorders; taking antipsychotics or benzodiazepines; not on a stable dose of antidepressant medication for at least 2 months; currently suicidal based on both self-reported diagnostic interview; currently receiving CBT for depression
Pagnini 2022[60]	United States of America	38	Non-CBT	5 weeks	Other (Amyotrophic lateral sclerosis)	D, A	HADS	Self-guided	Inclusion: None Exclusion: Significant uncontrolled psychiatric disease (for example, schizophrenia, bipolar disorder) in the opinion of the study neurologist
Peerani 2022[105]	Canada	101	CBT+	12 weeks	Other (Inflammatory bowel disease)	D, A	HADS	Personnel-facilitated Personnel: Masters students with motivational interviewing experience Facilitation: Once weekly phone call to support participation (followed script)	Inclusion: Elevated Perceived stress score ≥8 Exclusion: HADS depression >10; Perceived Stress Scale <7; new onset treatment for anxiety and depression within the past 3 months; psychosis
Pottgen 2018[90]	Germany	275	CBT+	12 weeks	Neurological	D, A	HADS	Self-guided	Inclusion: no major neurological or psychiatric comorbidities (dementia, stroke, autism, psychosis) Exclusion: None
Read 2020[97]	Australia	308	CBT	8 weeks	Multiple conditions	D, A	PHQ-9; GAD-7	Personnel-facilitated Personnel: Psychologist Facilitation: Weekly phone call or email to guide and promote adherence, answer questions, and provide feedback	Inclusion: None Exclusion: met criteria for minor/major depression; had a history of bipolar disorder or schizophrenia; had undergone psychological therapy in the 12-month period prior to recruitment
Rini 2015[61]	United States of America	113	CBT	8 weeks	Other (knee or hip osteoarthritis)	A	PASS-20	Self-guided	None
Rocamora Gonzalez 2022[95]	Spain	102	Non-CBT	4 weeks	Cancer	D, A	HADS	Self-guided	Inclusion: None Exclusion: Diagnosis of severe mental disorder according to DSM-5 with acute episode at the time of selection
Schröder 2014[91]	Germany	78	CBT+	9 weeks	Neurological	D	BDI-II	Self-guided	Inclusion: Depressive symptoms Exclusion: Without self-reported depressive symptoms, and with acute suicidal ideation; reporting diagnoses of psychosis or bipolar disorder, as well as suicidality, led to immediate exclusion
Schulz 2020[93]	Germany	118	CBT	6 weeks	Cardiovascular	D, A	HADS	Personnel-facilitated Personnel: Research team and clinical psychologist Facilitation: Discussion board moderated by clinical psychologist	Inclusion: At least mildly increased psychosocial distress (>6 points on the anxiety or depression subscale of the HADS or <16 points on the satisfaction with life scale) Exclusion: None
Van Beugen 2016[77]	Netherlands	131	CBT	25 weeks**	Other (Psoriasis)	D, A	BDI; ISDL	Personnel-facilitated Personnel: Research team and psychologist Facilitation: One to one orientation followed by weekly feedback on homework via the IMI platform	Inclusion: Positive psychological risk profile, i.e., Impact of Chronic Skin Disease on Daily Life score of ≥ 5 for anxiety and/or ≥ 21 for negative mood Exclusion: Psychological (i.e., diagnosis according to the Diagnostic and Statistical Manual of Mental Disorders (DSM)) and/or physical comorbidity interfering with the study protocol; current psychological treatment
Van Luenen 2018[76]	Netherlands	188	CBT+	8 weeks	Other (HIV)	D, A	PHQ-9; GAD-7	Personnel-facilitated Personnel: Masters psychology and science students Facilitation: Weekly 15-minute phone call to discuss content	Inclusion: Mild to moderate depressive symptoms (PHQ-9 score of >4 and <20) Exclusion: Severe depressive symptoms (PHQ-9 score ≥20); severe suicidal ideation (score >1 on the suicide item of the PHQ-9); receiving treatment from a psychologist or psychiatrist; had been on antidepressants for less than 3 months, or had changed type or dose of antidepressants in the past 3 months
Wadon 2021[85]	United Kingdom	20	CBT	8 weeks	Neurological	D, A	BDI; GAD-7	Self-guided	Inclusion: None Exclusion: Previous treatment with CBT; concurrent psychological therapy
Young 2021[49]	United Kingdom	125	CBT+	12 weeks	Other (Obesity)	D, A	PHQ-9; BDI	Self-guided	Inclusion: Current depressive symptoms indicated by a PHQ-9 score ≥5 Exclusion: Serious risk of suicide (determined via study psychologist); started a new antidepressant medication (or changed dose) in the past 4 weeks; started psychotherapy (or changed therapy arrangements) in the past 4 weeks
Younge 2015[78]	Netherlands	324	Non-CBT	12 weeks	Cardiovascular	D, A	HADS	Self-guided	None

Abbreviations: Non-CBT = Non-Cognitive Behavioural Therapy; CBT = Cognitive Behavioural Therapy (CBT); CBT+ = Cognitive Behavioural Therapy + non-CBT technique; HADS = Hospital Anxiety and Depression Scale; PHQ-8/9 = Patient Health Questionnaire 8/9; GAD-7 = Generalized Anxiety Disorder-7; DASS-21 = Depression, Anxiety and Stress Scale; CES-D = Center for Epidemiologic Studies Depression Scale; STAI = State-Trait Anxiety Inventory; BDI/BDI-II = Beck Depression Inventory; ASI = Anxiety Sensitivity Index; VSI = Visceral Sensitivity Index; MADRS-S = Montgomery-Asberg Depression Rating Scale; CAQ = Cardiac Anxiety Questionnaire; BAI = Beck Anxiety Inventory; ISDL = Impact of Chronic Skin Disease on Daily Life; ED-ASF 8a/ED-DSF 8a = Emotional Distress–Anxiety Short Form 8a / Emotional Distress–Depression Short Form 8a; DSM-5 = Diagnostic and Statistical Manual of Mental Disorders, 5^th^ Edition

*D = Depression; A = Anxiety

**Represents an average

### Risk of bias

The randomization procedure for most of the studies was considered adequate (38, 68%). Most studies reported details of allocation concealment (52, 93%), but the blinding of participants was well described in only a few studies due to the nature of the intervention (4, 7%). Most studies reported blinding of outcomes (50, 89%). Many studies had missing or incomplete outcome data due to participant drop out (44, 79%) (**S2 Appendix**).

### Harms

Eighteen of the studies included in this review reported on adverse events, including reporting zero events (n = 9) [52, 54, 55, 59, 61, 76, 83, 84, 87]. Of the nine studies that reported and collected data about adverse events, five reported physical and mental health events [66,72, 79, 86, 90], and four collected information related only to mental health events [49, 70, 71, 88] (i.e., worsening HADS-D scores, suicidality, etc.). Two of these studies provided specific thresholds for survey questionnaires, beyond which an adverse event was considered [71, 90]. When reported, the prevalence of adverse events ranged from 2% to 11%. Adverse events were listed as a primary or secondary outcome for two of the RCTs included in this review [86, 88]. For studies that provided this level of detail (n = 12), adverse events were collected by direct communication between participants and the study investigators [55, 59, 66, 72, 78, 79, 86], a questionnaire [49, 87, 88, 90], or a mixture of the two [54].

**Fig 2:**
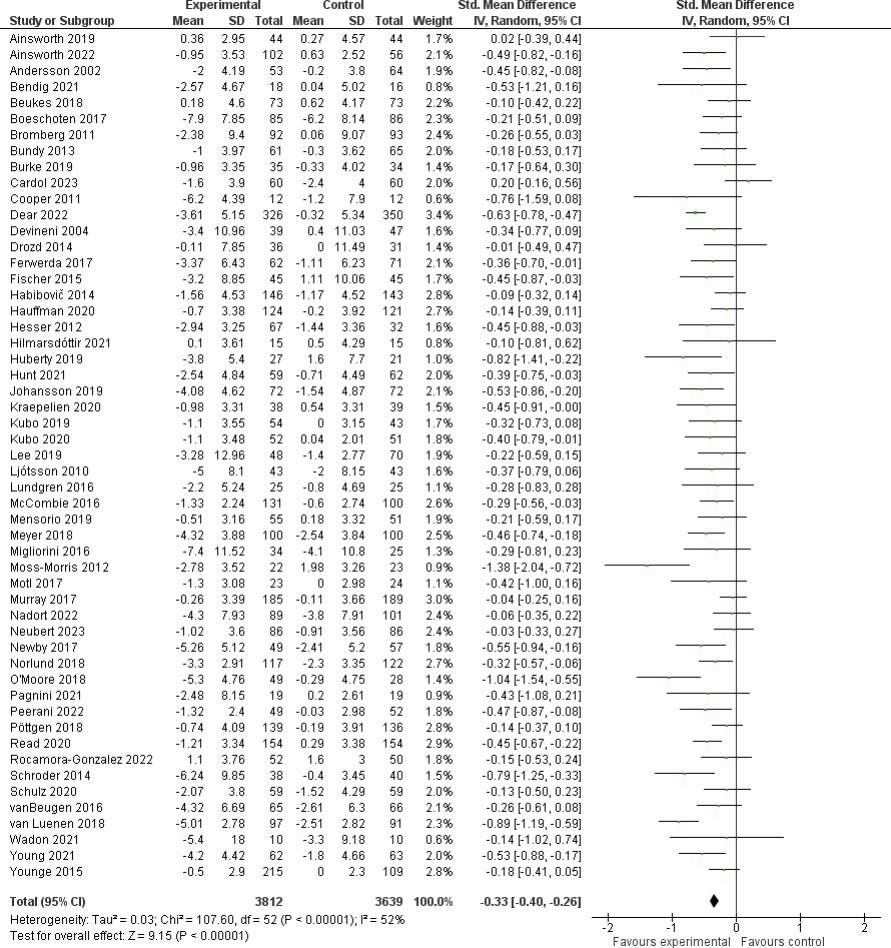
Effect of studies assessing depression.

Fig 2 depicts the forest plot showing the effect size of the change in depression between intervention and control groups. Depression was assessed in 53 studies with an overall sample size of 7463 participants. The results of the meta-analysis combining the 53 studies found a significant difference in change in depression scores between the intervention and control groups (*SMD* = -0.33 [-0.40, -0.26], p<0.001, I^2^ = 52%; **Fig 2**).

### Depression

Of eight measures used to report depression, the HADS-D was the most commonly reported (24, 45%), followed by the PHQ-8/9 (14, 26%). Subgroup analyses by the two most reported measures (HADS-D, PHQ-8/9) as compared to all other measures (**S3 Appendix**) showed no significant subgroup differences (p = 0.09), and a significant impact on depression scores in all subgroups. The greatest heterogeneity was in the PHQ-8/9 subgroup (I^2^ = 71%), followed by the HADS-D group (I^2^ = 24%). Subgroup analysis by intervention type (CBT, CBT+, and non-CBT interventions p = 0.35), personnel support level (“self-guided” versus “personnel-facilitated”, p = 0.88) and chronic disease groupings (p = 0.37) revealed significant impact on depression scores in all subgroups and no significant subgroup differences (**S4 Appendix to S6 Appendix**). The meta-regressions assessing participant mean age and intervention length with a change in depression score revealed a non-statistically significant relationship for either factor (intervention length: p = 0.37; age: p = 0.28) (**S7 Appendix)** supporting the beneficial impact on depression regardless of age or intervention length.

### Anxiety

**Fig 3:**
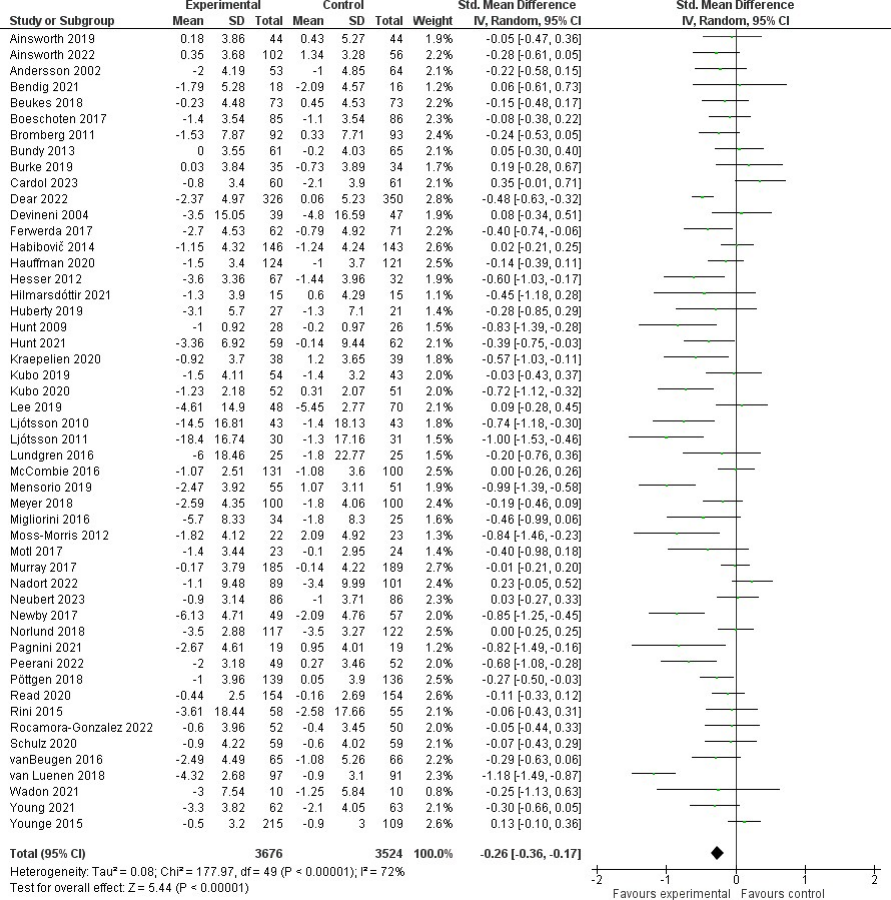
Effect of studies assessing anxiety.

A meta-analysis combining the results of 50 studies assessing anxiety with an overall sample size of 7211 participants showed a significant change in anxiety scores between intervention and control groups (*SMD* = -0.26 [-0.36, -0.17], p<0.001, I^2^ = 72%) (**Fig 3**).

Of 12 measures used to report anxiety, HADS-Anxiety (HADS-A) was the most common (24, 48%), followed by the GAD-7 scale (9, 18%). Subgroup analyses by the two most reported measures (HADS-A, GAD-7) as compared to all remaining measures (**S8 Appendix**) identified benefit across subgroups without subgroup differences (p = 0.30, I^2^ = 17%). Similarly, subgroup analyses of intervention type (CBT, CBT+, and non-CBT interventions, p = 0.09) and personnel support level (“self-guided” versus “personnel-facilitated, p = 0.53) showed (**S9 Appendix and S10 Appendix**) benefit across subgroups with no significant subgroup differences. For chronic physical condition groupings, although there was substantial heterogeneity (I^2^ = 82%), there were significant subgroup differences (p<0.001) identified. Significant impact on anxiety was seen in the neurological conditions (*SMD* = -0.26 [-0.38, -0.14], p<0.001) and ‘other’ subgroups (*SMD* = -0.34 [-0.50, -0.18], p<0.001), but the cardiovascular and cancer subgroups did not reach statistical significance (cardiovascular: *SMD* = 0.03 [-0.10, 0.15], p = 0.89, I^2^ = 0%; cancer: *SMD* = -0.18 [-0.39, 0.03], p = 0.08, I^2^ = 50%) (**S11 Appendix**). The results of the meta-regression assessing the impact of intervention length (p = 0.46) or age (p = 0.12) on anxiety were not significant (**S12 Appendix**) supporting beneficial impact on anxiety regardless of these factors.

### Adherence

Two types of adherence were described–intervention adherence and study adherence (i.e. study completion). Thirty-eight studies (68%) provided an explicit ‘dose’ or recommendation for how often the intervention should be completed to receive the most benefit [49, 50, 52–56, 58, 61, 62, 66–72, 74–76, 78, 80, 81, 85–87, 89, 90, 92–96, 99–101, 104–106]. Nearly all studies described intervention adherence (43, 77%) [49, 51, 54, 55, 57–59, 61, 63–72, 74–80, 82–86, 90, 92, 94–96, 98–101, 103, 105], including homework/task/lesson completion (11, 20%)[55, 65–68, 71, 72, 77, 82, 96, 103], user data as tracked by the platform (logins, video completion) (19, 34%) [51 54,57,58,69,70,74,77–79,83–85 90,94,95,99,101,105], and self-report (13, 23%)[59, 61, 75, 76, 80, 83, 88, 91–93, 97, 98, 100] methods. When using homework/tasks completed in studies where data was reported (n = 3) [67, 71, 77], intervention adherence ranged from 69% to 92%. For these studies, participants were more commonly divided according to level of tasks completed (e.g., non-completers vs completers) than as continuous data (e.g., proportion that completed week 1, week 2, etc.). Study adherence was provided by all studies and defined as end-of-study survey completion/dropouts. When defined as end-of-study survey completion, and when data were reported (n = 4) [59,77,80 92], study adherence ranged from 73% to 97%.

## Discussion

This systematic review and meta-analysis of 56 RCTs, involving 7691 individuals with chronic physical conditions, found that scalable mind-body IMIs significantly improved symptoms of depression and anxiety compared to control conditions. Cognitive behavioral therapy was the primary approach in 50% of trials and was combined with non-CBT interventions in 32% of trials. Effect sizes for depression (*SMD* = -0.33 [-0.40, -0.26], p<0.001) and anxiety (*SMD* = -0.26 [-0.36, -0.17], p<0.001) were statistically significant and moderate in magnitude. These effect sizes are consistent with those from another review comparing face-to-face CBT to IMI CBT[107], and a review by Tao and colleagues (2023) focusing solely on CBT-based IMIs (depression SMD = -0.45; anxiety SMD = -0.33) [24]. Notably, the effect sizes in this review are also comparable to pharmacotherapeutic approaches for anxiety [108] and depression[109, 110].

This review is unique in its emphasis on scalable self-guided (45%) or minimally supported (55%) mind-body IMIs. In this review, small but statistically significant pooled effect sizes were observed, with no discernible difference between the self-guided and limited personnel support subgroups. This benefit despite level of personnel support is different from what was hypothesized but is a favorable conclusion in that the associated costs of a personnel supported approach may limit widespread implementation. Notably, as this review was focused on no or minimal personnel support, the impact of more intensive personnel support could not be evaluated. Moreover, the results can only be generalized to the population in which the intervention was evaluated. As detailed in Table 1, multiple studies excluded those participants who may have necessitated additional personnel support, including those with suicidal ideation and co-morbid psychiatric conditions.

The review, which includes CBT, non-CBT, and combination interventions found no significant differences in the impact on mental health outcomes between these subgroups. While there were a relatively small number of trials involving solely non-CBT interventions (10 trials, 18%) and some statistical heterogeneity mandating cautious interpretation, it is noteworthy that other published data align with these findings of similar mental health outcomes with both CBT and non-CBT interventions. For instance, a systematic review of 30 RCTs, four of which used online platforms, revealed the equivalence of CBT and mindfulness-based interventions in terms of depression [111]. Another systematic review and meta-analysis of 30 RCTs found no differences in depression outcomes between a CBT-based intervention, physical exercise, or a combination of the two [112]. The body of evidence supporting the impact of non-CBT interventions on mental health outcomes continues to grow, encompassing mindfulness-based stress reduction, other meditation techniques, and mindful movement [113–115]. Additionally, studies on breathwork practices have shown a small-medium effect in reducing stress, anxiety, and depression in clinical populations [23, 116–120]. The findings of the current review lend support to the idea that non-CBT and combination interventions can serve as alternative strategies to promote mental health [111]. This is of particular relevance as CBT, while recommended in clinical practice guidelines to manage anxiety [121] and depression [122], may not be effective for all patients. Future studies can add to the understanding of how best to tailor the choice of mind-body IMI to individual needs. In the meantime, it has been suggested [10] that psychotherapeutic techniques like CBT may be most effective in people with primary mental health conditions [123], whereas traditional mind-body techniques like yoga and Tai-chi may be most effective for mental health concerns in those living with physical health conditions [124, 125]. As 10–36% of people living with chronic physical conditions also live with clinical depression or anxiety, it is anticipated that a combination of both practices may prove to offer the most variety to participants and be the most applicable [126, 127].

Contrary to initial hypotheses, the age of participants in the review did not significantly influence depression or anxiety outcomes, despite potential variations in how different age groups engage with IMIs. The “digital divide” [128]—which represents a gap in technology access and use [129] can create barriers to healthcare [130]. Unfortunately, few studies included in this review provided information on participants’ technological literacy or socioeconomic status beyond race, marital status, and sex. To address technology access disparities, future studies should collect baseline data on socioeconomic status and technological literacy. A 2021 systematic review of digital interventions for physical activity showed benefits for individuals of high socioeconomic status, but not for individuals of lower socioeconomic status [131]. The extent to which these findings are mimicked in mind-body IMIs has yet to be evaluated.

Within the limitations of moderate heterogeneity, the review discovered that mind-body IMIs had varying effects on different chronic conditions. While benefit was seen for symptoms of depression across all chronic condition subgroups, anxiety symptoms were not impacted in the cancer and cardiovascular subgroups. It is unclear whether this is a true finding, or whether it is instead related to insufficient data to draw conclusions. In the anxiety related analysis, there were a small number of studies in the cancer (n = 6) and cardiovascular groups (n = 6), alongside notable heterogeneity in the clinical populations enrolled. Across studies amongst individuals with cancer, differing tumor stages were enrolled [54, 57, 58, 64]. In the cardiovascular subgroup, enrollment ranged across individuals with a recent myocardial infarction [70], those with established coronary artery disease [65, 69, 92], and those who received an implantable cardiovascular device [74, 93]. The experience [132] and prevalence [23] of anxiety may be influenced by disease stage [133, 134], and factors like sex, location, and socioeconomic status [6, 126, 133]. This study points to the need for additional research evaluating the role of IMIs for the management of anxiety in these subgroups.

The current review aimed to assess intervention adherence but faced challenges due to limited reporting and inconsistent adherence definitions. Adherence is a crucial measure of the success of mind-body interventions. In a >200,000 patient systematic review of app-based interventions for chronic disease management, IMIs experience dropout rates as high as 43% [135]. From other reports, up to 80% of enrolled participants have been found to engage minimally [136, 137], with fewer than 5% being daily users in real world settings [138, 139]. Studies included in the current review lacked consistency on the measurement of adherence. Survey completion and self-reports were the most common markers, despite their limitations like recall bias, overestimation, and the possibility that participants may feel clinical improvement and stop using the intervention [140]. Adherence is most useful in explaining outcomes when interventions have a lower effect than hypothesized, making it an important factor for future studies to document carefully. Future research should explore alternative data collection methods, including tracking of activity within the IMI or the use of sensors or physiological measures for more objective evaluation.

While this study had strengths, including the inclusion of RCTs, >7500 participants, and diverse chronic conditions, there are several limitations to consider. Moderate to substantial heterogeneity [141] and variations in interventions and clinical characteristics were observed among the included studies. Effect sizes for anxiety and depression were combined within four chronic physical condition subgroups, acknowledging potential disparities in symptom experience and impact. To mitigate variability, a random effects standardized mean difference model was used to calculate the pooled difference. Missing mean change score data in some studies required manual calculation [47, 142], potentially introducing inaccuracies. When unavailable, authors were contacted via email for their data (n = 10; 4 responded). Variations in eligibility thresholds for validated questionnaires could lead to bias or dilution of treatment effects. As detailed in Table 1, mental health inclusion and exclusion criteria differed across studies, with varying cut-points for eligibility and variable exclusion of individuals with psychiatric comorbidities and suicidality. Lastly, the evidence base had a geographical bias toward high-income countries, limiting the generalizability of the findings to low- and middle-income countries.

## Conclusions and future directions

In an era of a growing reliance on digital health and rising rates of chronic disease and mental distress, this meta-analysis of RCTs reveals that scalable mind-body IMIs have a statistically significant impact on depression and anxiety symptoms across various chronic physical conditions. To further the understanding of the magnitude of benefit and generalizability, large-scale transdiagnostic RCTs are welcomed that include participants with varying chronic physical conditions, collect factors associated with technology proficiency, and assess cost-effectiveness. Additional focus is also required on the effect of non-CBT based IMI interventions. Awaiting further advancements in this field, and under the weight of growing chronic disease and mental distress, the evidence synthesized by this review is sufficient to assist healthcare providers and policymakers to lobby for these strategies as accessible and effective adjuncts to clinical care.

## Supporting information

S1 Appendix Search Strategies. (DOCX)

S2 Appendix Risk of Bias. (TIF)

S3 Appendix Depression subgroup analyses by scale. (TIF)

S4 Appendix Depression subgroup analyses by intervention type. (TIF)

S5 Appendix Depression subgroup analyses by delivery modality. (TIF)

S6 Appendix Depression subgroup analyses by chronic condition groupings. (TIF)

S7 Appendix Effect of intervention length and age on standardized mean change in depression score. (TIF)

S8 Appendix Anxiety subgroup analyses by scale. (TIF)

S9 Appendix Anxiety subgroup analyses by intervention type. (TIF)

S10 Appendix Anxiety subgroup analyses by intervention delivery modality. (TIF)

S11 Appendix Anxiety subgroup analyses by chronic condition groupings. (TIF)

S12 Appendix Effect of intervention length and age on standardized mean change in anxiety score. (TIF)

S13 Appendix PRISMA Checklist. (DOCX)
