# Supplementary figures and images for: Mind-body internet and mobile-based interventions for depression and anxiety in adults with chronic physical conditions: A systematic review of RCTs
Source: PLOS Digit Health. 2024 Jan 23;3(1):e0000435. doi: 10.1371/journal.pdig.0000435 (PMC10805319; doi:10.1371/journal.pdig.0000435)

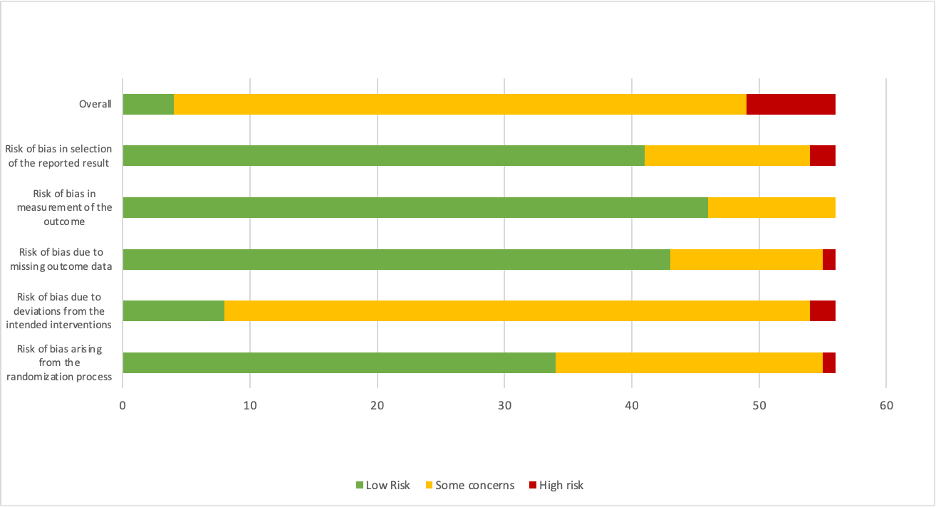

Supplement: S2 Appendix — (TIF) [file pdig.0000435.s002.tif]

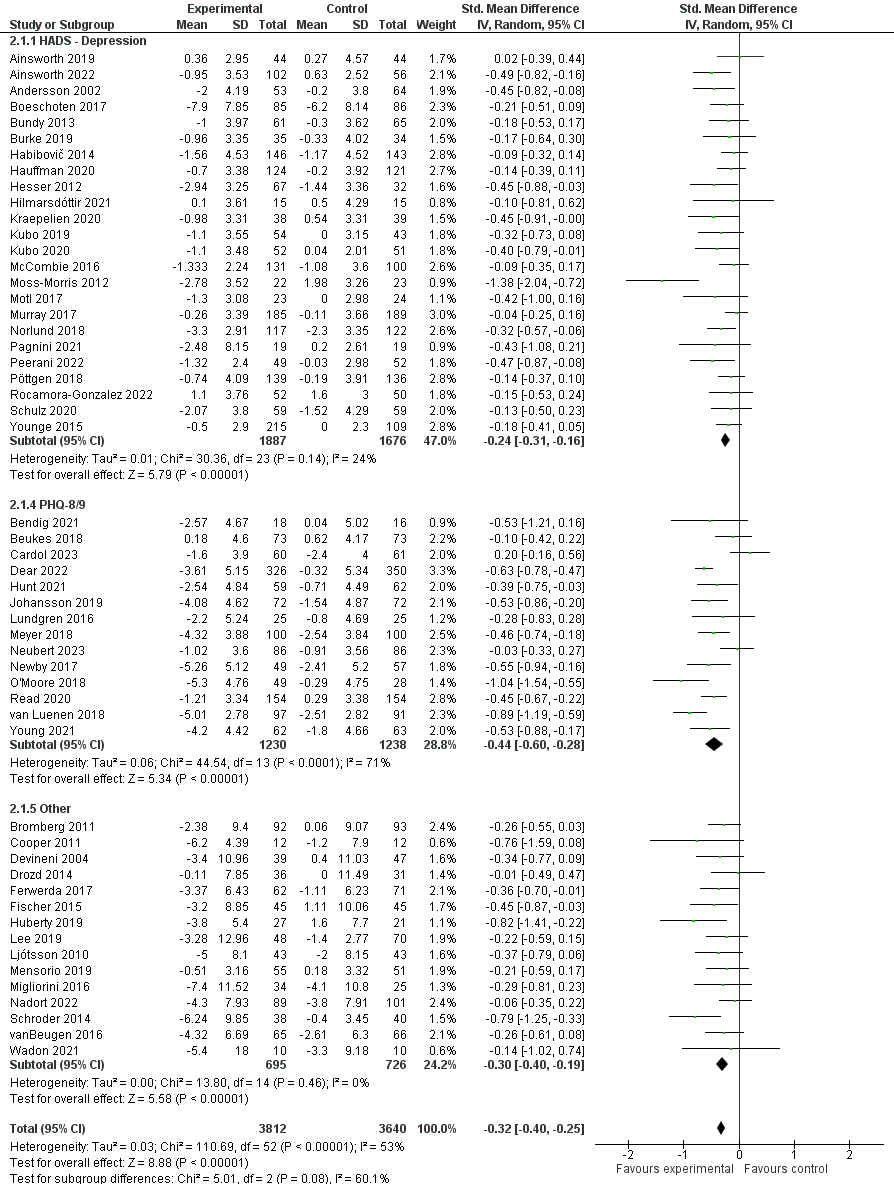

Supplement: S3 Appendix — (TIF) [file pdig.0000435.s003.tif]

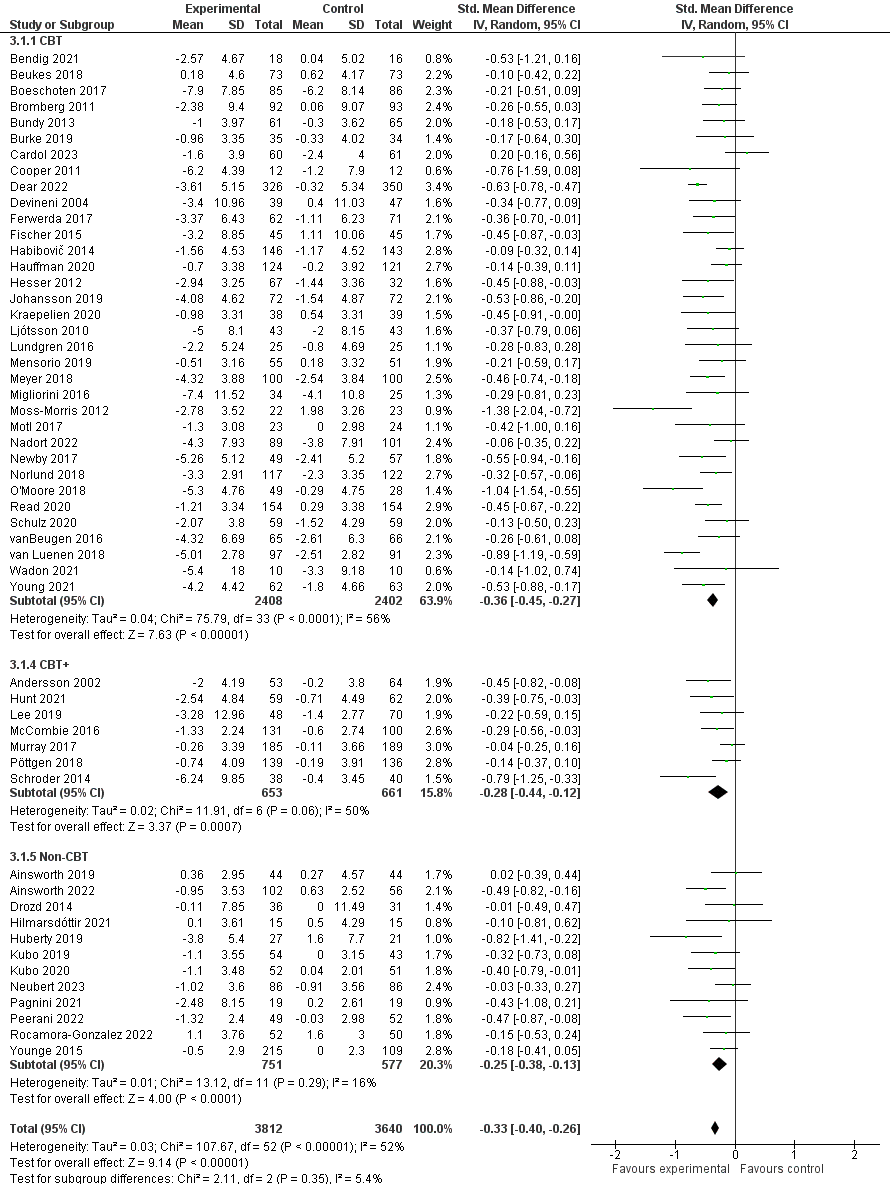

Supplement: S4 Appendix — (TIF) [file pdig.0000435.s004.tif]

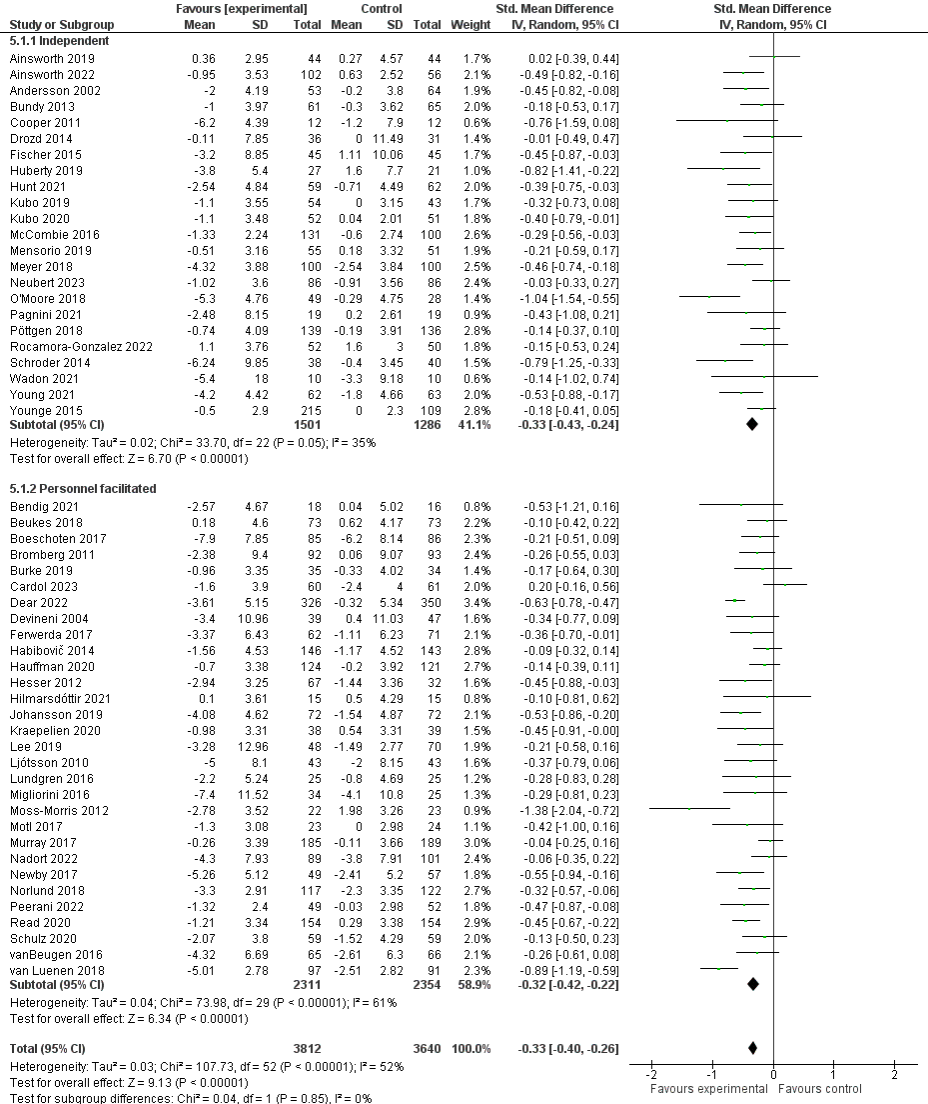

Supplement: S5 Appendix — (TIF) [file pdig.0000435.s005.tif]

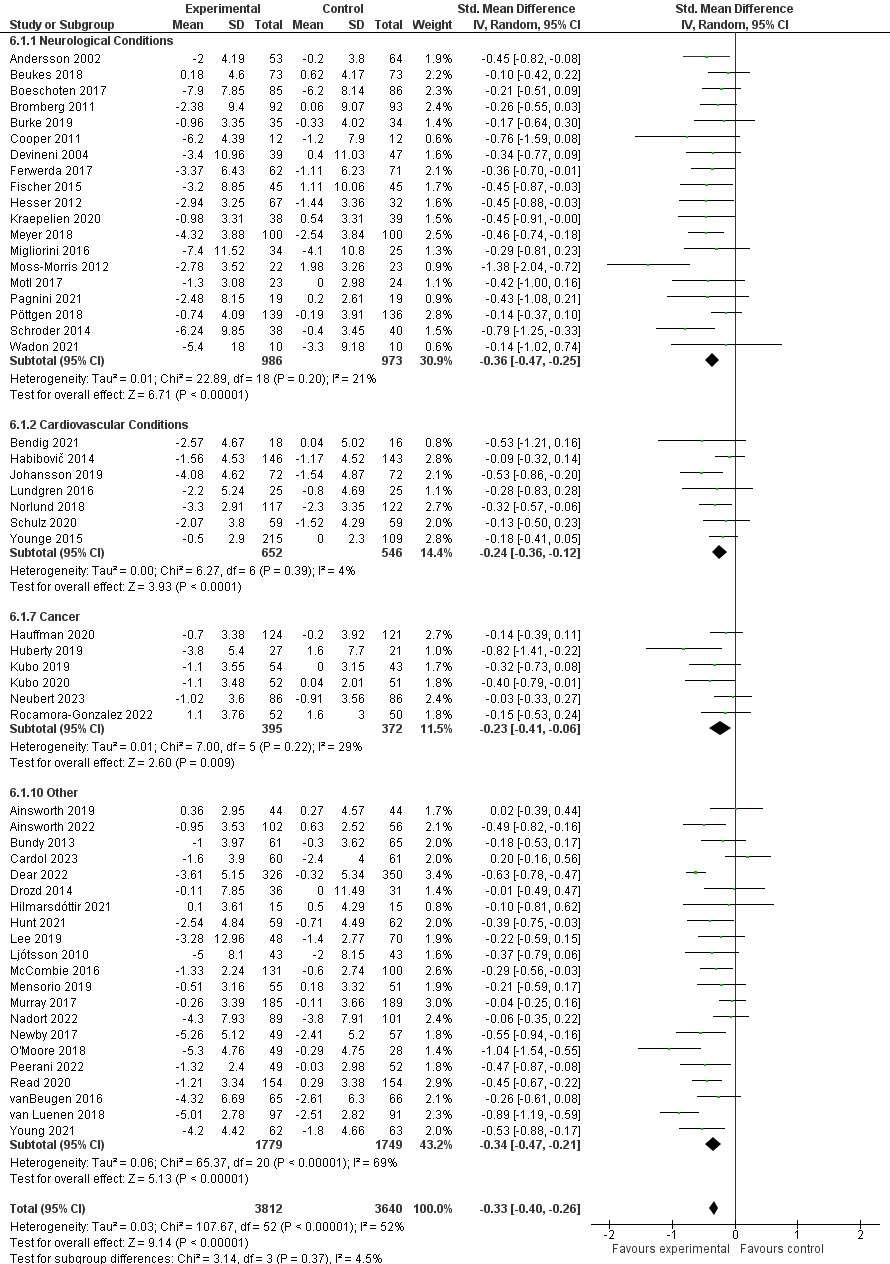

Supplement: S6 Appendix — (TIF) [file pdig.0000435.s006.tif]

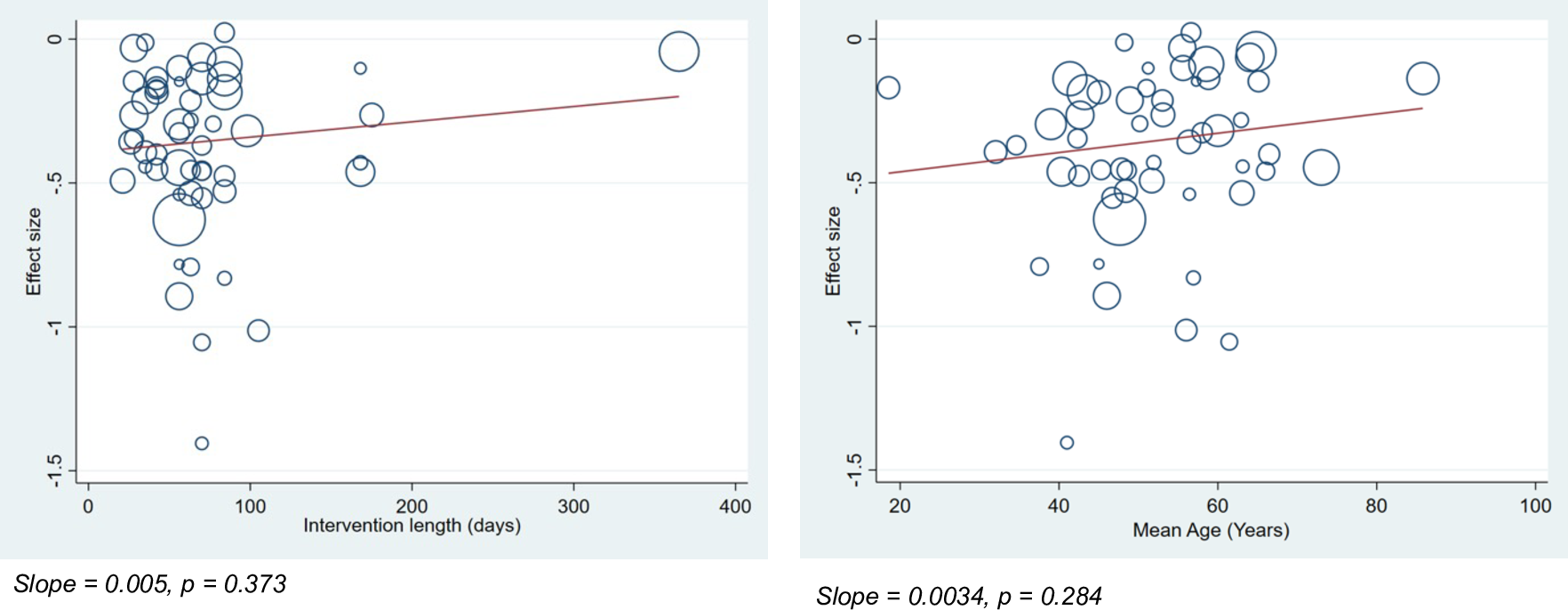

Supplement: S7 Appendix — (TIF) [file pdig.0000435.s007.tif]

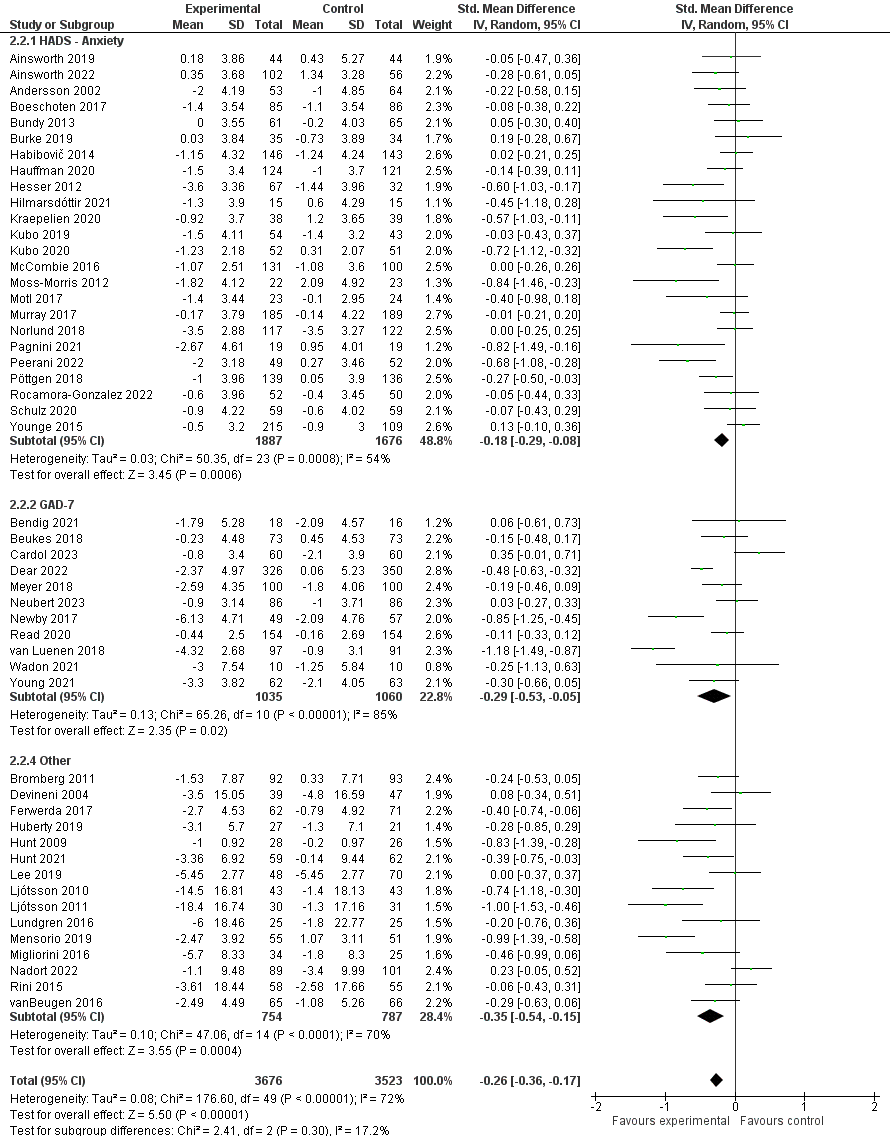

Supplement: S8 Appendix — (TIF) [file pdig.0000435.s008.tif]

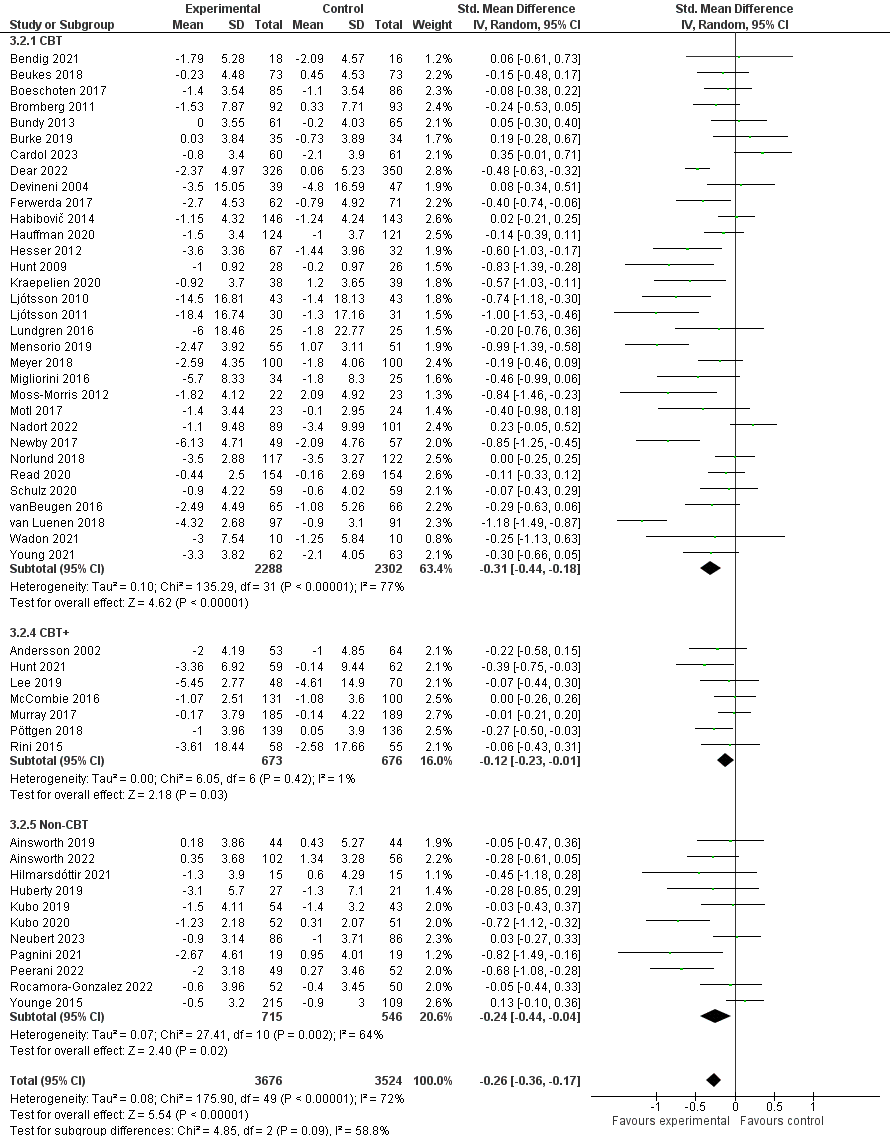

Supplement: S9 Appendix — (TIF) [file pdig.0000435.s009.tif]

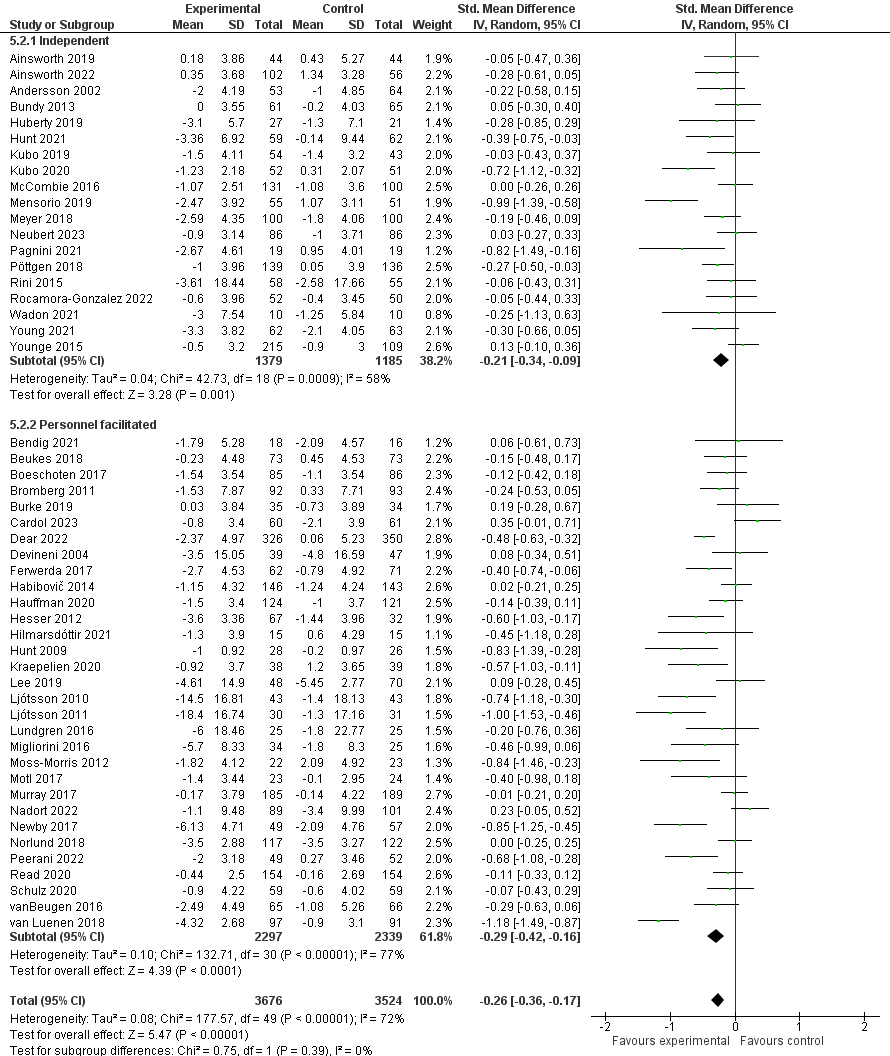

Supplement: S10 Appendix — (TIF) [file pdig.0000435.s010.tif]

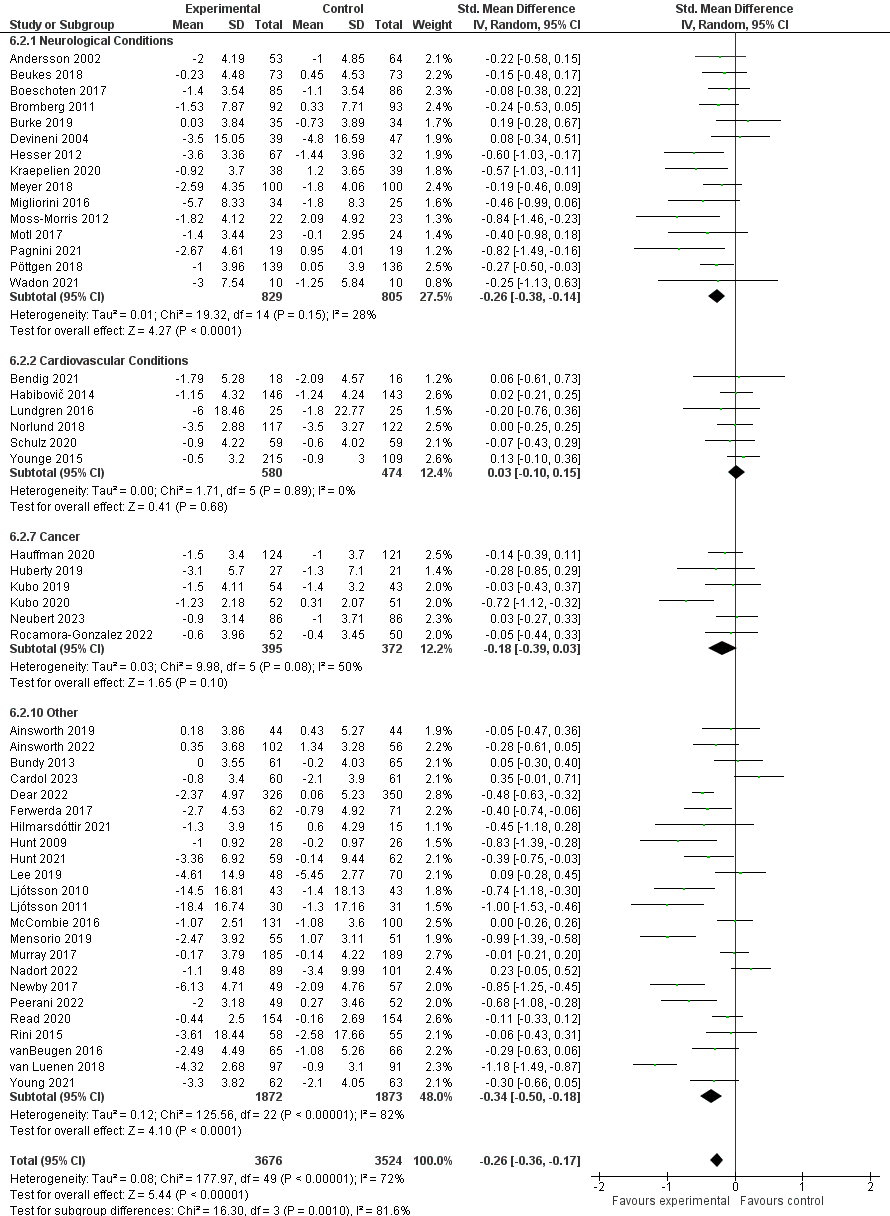

Supplement: S11 Appendix — (TIF) [file pdig.0000435.s011.tif]

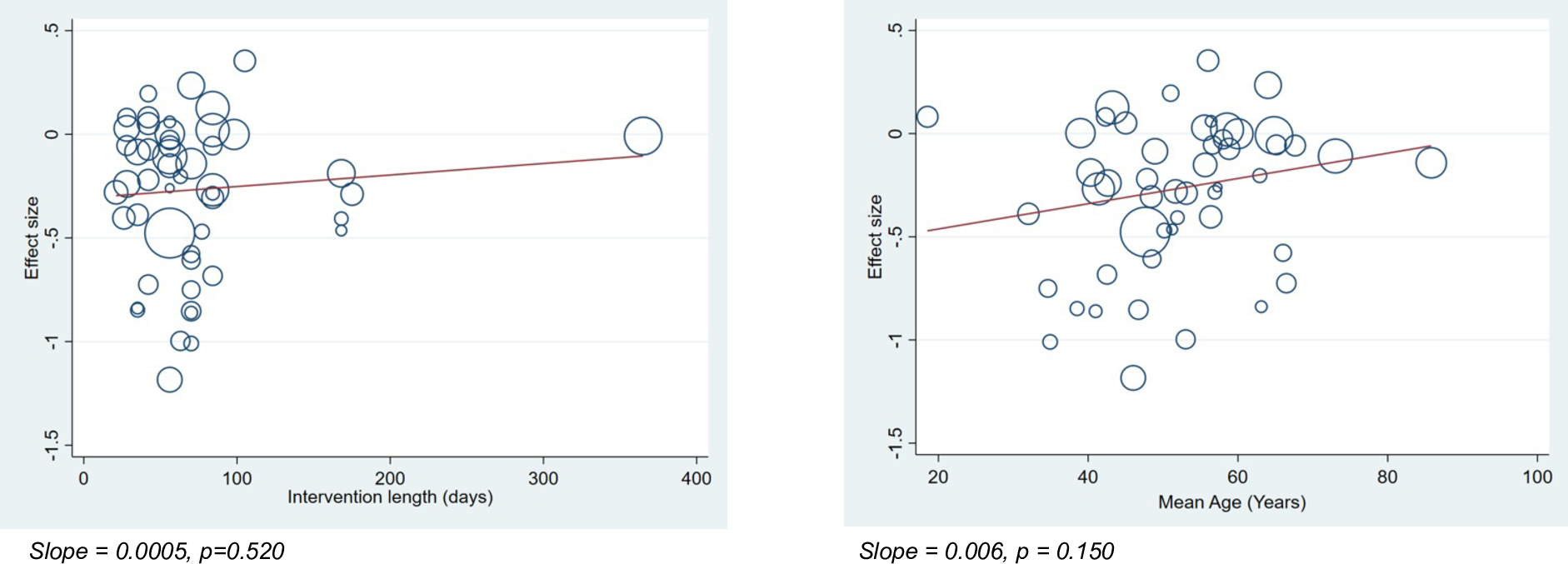

Supplement: S12 Appendix — (TIF) [file pdig.0000435.s012.tif]
